# Modeling and Visualizing Cell Type Switching

**DOI:** 10.1155/2014/293980

**Published:** 2014-04-14

**Authors:** Ahmadreza Ghaffarizadeh, Gregory J. Podgorski, Nicholas S. Flann

**Affiliations:** ^1^Computer Science Department, Utah State University, Logan, UT 84322, USA; ^2^Biology Department, Utah State University, Logan, UT 84322, USA; ^3^Center for Integrated BioSystems, Utah State University, Logan, UT 84322, USA; ^4^Institute for Systems Biology, Seattle, WA 98109, USA; ^5^Synthetic Biomanufacturing Institute, Logan, UT 84322, USA

## Abstract

Understanding cellular differentiation is critical in explaining development and for taming diseases such as cancer. Differentiation is conventionally represented using bifurcating lineage trees. However, these lineage trees cannot readily capture or quantify all the types of transitions now known to occur between cell types, including transdifferentiation or differentiation off standard paths. This work introduces a new analysis and visualization technique that is capable of representing all possible transitions between cell states compactly, quantitatively, and intuitively. This method considers the regulatory network of transcription factors that control cell type determination and then performs an analysis of network dynamics to identify stable expression profiles and the potential cell types that they represent. A visualization tool called *CellDiff3D* creates an intuitive three-dimensional graph that shows the overall direction and probability of transitions between all pairs of cell types within a lineage. In this study, the influence of gene expression noise and mutational changes during myeloid cell differentiation are presented as a demonstration of the *CellDiff3D* technique, a new approach to quantify and envision all possible cell state transitions in any lineage network.

## 1. Introduction

During development, a complex system of tissues and organs emerges from a single cell by the coordination of cell division, morphogenesis, and differentiation. Understanding the differentiation of cell types is necessary to understanding development and its associated defects, for improved control of stem cell differentiation in therapeutic use and for taming diseases such as cancer. Cellular differentiation occurs when a less specialized cell or its progeny becomes increasingly specialized by acquiring properties that allow specific functions. In animals, differentiation typically results in a terminally differentiated state in which a specialized cell can no longer acquire the properties of other specialized adult cells. Recent discoveries, however, have shown that terminally differentiated cells can be reprogrammed to revert back to multipotent and pluripotent stem cells which have the potential to differentiate into other cell types [1, 2] or to transdifferentiate into other specialized cell types [3].

**Figure 1:**
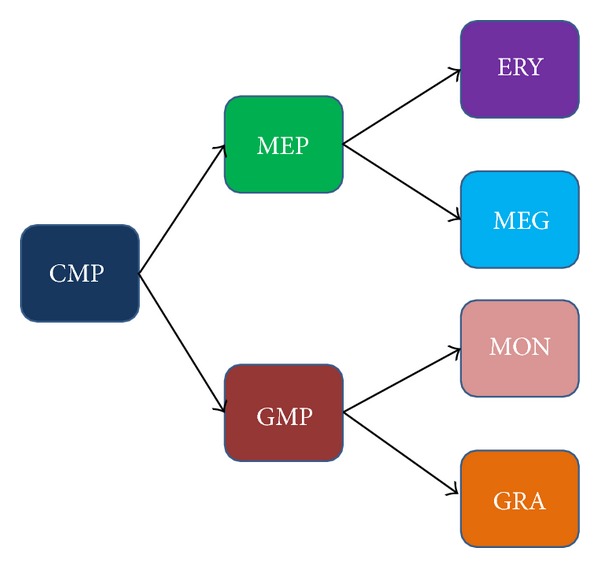
A simplified myeloid lineage tree from [4] where the terminal nodes are mature terminally differentiated erythrocytes (ERY), megakaryocytes (MEG), monocytes (MON), and granulocytes (GRA). Multipotent cells are the common myeloid progenitor (CMP), megakaryocyte-erythrocyte progenitor (MEP), and granulocyte-monocyte progenitor (GMP). The color assigned to each cell type in this figure is also used in the differentiation network shown in Figure 4.

Differentiating cells normally follow well defined paths to mature cell types. Taken together, these paths are referred to as a lineage tree. Pluripotent stem cells give rise to progeny that specialize into more constrained multipotent cells. In turn, multipotent cells produce a variety of stable, terminally differentiated cells. This process is usually depicted as a tree with a pluripotent cell at its root, multipotent cells as intermediate nodes, and the mature cell types as branch tips. As an example, a simplified portion of the myeloid cell lineage tree is illustrated in Figure 1. This figure shows that common myeloid progenitor stem cells produce two pluripotent cell types, a megakaryocyte-erythrocyte progenitor and a granulocyte-monocyte progenitor, that in turn produce terminally differentiated erythrocytes, megakaryocytes, monocytes, and granulocytes.

Intracellular genetic regulatory networks (GRNs) control differentiation by responding to external (extracellular) and internal (intracellular) stimuli that reconfigure gene expression profiles and change cell physiology [5]. There is a growing body of evidence that cell types are determined by stable expression patterns of the regulatory networks, referred to as attractors. Switching between cell types amounts to transitioning from one attractor to another [6]. The attractor model explains how cell types can be stable under gene expression noise and how changes in the expression of a small number of master regulators can shift the expression of hundreds of genes as cell types switch.

Regulatory network dynamics are driven by molecular events within the cell that are subject to noise [7]. Understanding the role of noise in gene expression and its effect on differentiation is essential to gaining insight into cellular specialization and its errors. If cell types are attractors of the GRN, these attractors must be robust to noise in order to maintain particular cell types and to stay on the correct branches of the lineage tree during differentiation. Failure to do either can have dire consequences. For instance, cancer has been proposed to involve destabilization of attractor states due to changes in genetic regulatory network dynamics [8]. In this view, the attractors that correspond to normal cells switch to new, abnormal attractors characteristic of cancer cells. In addition to pathological states, transitions between attractor states of differentiated cells may lead to dedifferentiation, in which a cell reverts to an earlier multipotent state, or transdifferentiation, in which a differentiated cell switches to another adult differentiated cell type [9]. Abnormal type switching may also result in off-differentiation in which a multipotent cell from one branch of a lineage tree is converted to a differentiated cell on another branch of the tree. Finally, to maintain a population of multipotent cells, at least some of these cells must resist differentiation to later stages within the lineage tree [10].

**Figure 2:**
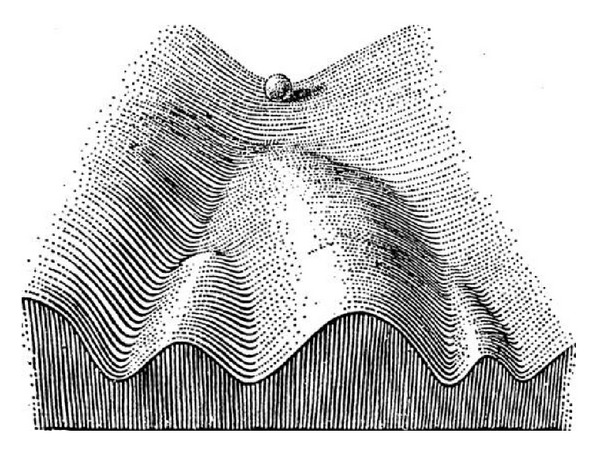
Waddington's classic model of an epigenetic landscape [11]. A developmentally immature cell, represented as a ball at the top rolls downhill and is deflected right or left at each branch point until it reaches a catch basin (not shown in this diagram) that corresponds to a terminally differentiated cell.

An early and influential way of viewing differentiation is Waddington's [11] epigenetic landscape. Waddington envisioned differentiation occurring on a rugged landscape of sloping ridges and valleys (see Figure 2). Waddington represented an undifferentiated cell as a ball at the uppermost point of the highest valley. Differentiation occurred as this ball rolled downhill, encountering the ends of ridges that define branch points between valleys. At each of these branch points, the ball moved left or right to follow the new sloping valley to another ridge terminus that separates yet another pair of valleys. Each ridge terminus represents a progenitor cell in a conventional lineage tree and the movement to right or left into a new valley from this branch point represents a commitment of the progenitor to one or another lineage. The ridges represent barriers that maintain a cell state once it is chosen.

In the decades since Waddington proposed his model, many investigators have used the concept of an epigenetic landscape and tailored it to explain a variety of developmental processes. Waddington himself cautioned that the epigenetic landscape is an abstraction that could not be rigorously interpreted [11]. Some recent work has tried to enhance Waddington's epigenetic landscape to move it from metaphor to rigorous model [1, 12–15]. However, even with these extensions, the ridge-and-valley topography of the epigenetic landscape places a fundamental limit on the number and kinds of cell type transitions that can be shown. For example, representing transdifferentiation between nonadjacent lineages in Waddington's model requires jumping over two or more ridges and showing dedifferentiation requires uphill movement. Conventional two-dimensional lineage trees suffer from similar problems. Even more significant than difficulties in visually representing nonstandard, yet documented transitions between cell types is that Waddington's epigenetic landscape and conventional lineage trees both fail to provide quantification of the probability of any transition. Finally, epigenetic landscapes and conventional lineage trees show only a small fraction of the possible transitions between cell types. Many of these transitions were previously considered hypothetical, but with ability to induce pluripotent stem cells from adult differentiated cells and to induce transdifferentiation between lineages, these changes in cell type are well known. To illustrate the limitations of standard representations of cell lineages, a generalized epigenetic landscape like that shown in Figure 2 that considers *m* cell type attractors can only represent a maximum of 2*m* − log⁡_2_(*m* + 1) − 1 cell type transitions. This formulation considers the expected differentiation transitions within the lineage tree (*m* − 1) and transdifferentiation events between adjacently arranged cell types on the tree (*m* − log⁡_2_(*m* + 1)). As the number of cell types in a system increases, the limitations of the epigenetic landscape become more acute: the number of representable transitions grows with *O*(*m*), while the number of possible transitions grows with *O*(*m* ^2^). Given that nonstandard attractor type transitions play key roles in cancer and disease development, coupled with the ability to experimentally induce dedifferentiation and transdifferentiation and the possibility of off-differentiation events, improvements are needed in the visualization of cellular differentiation.

In this work, we present a new method that generates a three-dimensional graph of attractors and all possible transitions between them to overcome the limitations of a conventional representation of cellular differentiation. Our technique, implemented by a tool called* CellDiff3D*, analyzes the network of attractors generated by a random Boolean GRN. In this work, the GRN that simulates myeloid cell differentiation is used as a demonstration. A noise analysis of the network dynamics is performed to identify *m* attractors and the likelihood of all the possible *m*(*m* − 1) transitions between them. This information determines the layout of the graph. The graph is easy to interpret and qualitatively represents the likelihood of transitions between cell types, their overall direction, and rate under the influence of noise. Visualization of the results of* CellDiff3D* is achieved by virtual reality modeling language (VRML) that allows the user to zoom and rotate the three-dimensional lineage network. The* CellDiff3D* tool can be downloaded from http://www.celldiff3d.org/.

## 2. *CellDiff3D* Design and Visualization

### 2.1. Separation and Flux between Attractors

We use the mean first passage time (MFPT) [16] between the attractors of any given GRN, represented qualitatively as a Boolean network [17]. MFPT determines the probability and directionality of each theoretically possible transition between all pairs of network states. Introduced by Shmulevich et al. [16], MFPT(*a*_*i*_, *a*_*j*_) between a pair of attractors, *a*_*i*_ and *a*_*j*_, is an estimate of the average number of state update steps of a Boolean network that are required to transition from an attractor state *a*_*i*_ to an attractor state *a*_*j*_ when the network operates under uniform random noise. Noise is modeled by having each bit (gene expression value) have a probability of changing states (a bit flip, from expressed to nonexpressed or vice versa) at each state update step. Low MFPTs indicate a high likelihood of a transition between cell states and high MFPTs indicate low likelihood for this transition. Once MFPT between two attractors of a network is estimated, then two useful derived measures of the epigenetic barrier between attractors can be determined: the separation between attractors and the flux of transitions between them. Let the separation between two attractors *i*, *j* be

(1) $$\begin{array}{c} \text{separation}\left(i,j\right)=\min\left(\text{MFPT}\left(i,j\right),\text{MFPT}\left(j,i\right)\right). \end{array}$$

Higher separation implies a lower likelihood of transition between attractors. Note that separation is symmetric. Flux establishes the directionality of the transition by quantifying the difference between the rates (MFPTs) of forward and reverse transitions between a pair of attractors. The flux between attractors *i*, *j* is defined as

(2) $$\begin{array}{c} \text{flux}\left(i,j\right)=\text{MFPT}\left(i,j\right)-\text{MFPT}\left(j,i\right). \end{array}$$

Note that flux establishes overall direction of the transition between cell states and is asymmetric.

### 2.2. Network Dynamics Visualization

An important element of GRNs is their behavior under gene expression noise. By definition, attractors are stable expression states of a genetic regulatory network, but this stability is relative and expected to vary depending on the network structure and dynamics. For example, terminally differentiated cell states are expected to be more stable than progenitor cells that may be more sensitive to noise-driven changes in states. High levels of gene expression noise may cause unexpected or pathological cell state transitions, with these transitions categorized based on the relative positions of the source and sink cell types in the normal lineage tree.  Table 1 summarizes five kinds of transitions between cell types and provides an example of each case with respect to the cell types in the simplified myeloid lineage tree shown in Figure 1.

Two of these five transition types are represented easily in Waddington's epigenetic landscape: differentiation (moving “downhill” in the landscape toward more specialized cell types) and dedifferentiation (loss of specialization shown by upward movement). Two other transition types cannot be shown in the classic epigenetic landscape representation: off-differentiation (differentiation to a cell type not on the normal lineage path); and off-dedifferentiation (loss of specialization to a cell type off the normal lineage path). Additionally, the epigenetic landscape limits visualization of transdifferentiation events (a switch from one adult differentiated cell type to another) to only those events that occur between adjacently arranged cell types. As discussed earlier, it is important to have a way of representing all possible transition types because off-differentiation and dedifferentiation are likely to play central roles in cancer [3, 8] and because recent evidence suggests that transdifferentiation may occur during normal development [18] as well as being induced in cultured cells [19].

Our method visualizes the different attractor transition kinds by constructing a 3-dimensional graph in which the distances between pairs of cell types are their separation (the minimum MFPTs between each pair) and the favored direction of the transition is shown by an arrow with a thickness proportional to the flux. In this way, the graph provides a quantitative view of these important parameters. To reach this result, the following steps are taken. First, the attractors of a given network are determined. Next, noise analysis (described later) is performed for each attractor pair and the separation and flux values are calculated. This is followed by mapping separation and flux values to a weighted directional graph in which attractors are shown as nodes. Mapping is done using* Graphviz*, an open source graphing application [20]. All these procedures are described in detail in the Methods Section below. Plotting separation and flux values using* Graphviz* produces 3-dimensional layouts of the graph which can be rotated freely in any web browser and that are easy to understand and analyze.

The graphical layout problem for showing cell type switching is defined in the following way. Let *i*_*x*,*y*,*z*_ be the 〈*x*, *y*, *z*〉 coordinate of attractor *i* in the graph visualization, and let dist(*i*, *j*) be the Euclidean distance between points *i*_*x*,*y*,*z*_ and *j*_*x*,*y*,*z*_. Then, given a graph of *m* attractors defined as a set of separation  (*i*, *j*) | 1 ≤ *i*, *j* ≤ *m*, the layout is defined by determining the set of coordinates for each attractor such that the following summation is minimized:

(3) $$\begin{array}{c} \underset{1\leq i,j\leq m}{\sum }{\left(\mathrm{dist}\left(i_{x,y,z},j_{x,y,z}\right)-\text{separation}\left(i,j\right)\right)}^{2}. \end{array}$$

After determining the location of attractors (nodes) in 3D space, flux between pairs of attractors is represented by arrows (directed edges) of variable width between them with arrow width proportional to flux. The edge direction is given by the relationship between MFPT(*i*, *j*) and MFPT(*j*, *i*): if MFPT(*i*, *j*) < MFPT(*j*, *i*), then the edge is from *i* to *j*. The 3D graph is viewable in any web browser using the VRML viewer plugin (such as* Cartona3D*) and allows the user to rotate and zoom the graph to aid viewing, analyzing, and understanding the relationships between attractors within complex networks.

### 2.3. Visualizing the Myeloid Differentiation Network

**Figure 3:**
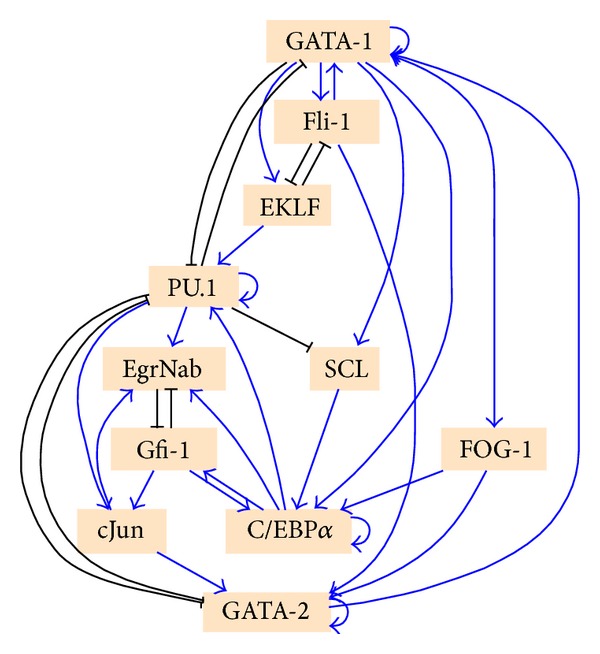
The genetic regulatory network used in this work for modeling myeloid differentiation. Nodes are eleven transcription factors that control cell lineage and edges are regulatory interactions between the transcription factors. An arrow signifies activation and a closed line signifies inhibition. The Boolean regulatory control functions are not shown. This network was discovered using a new search algorithm (paper in preparation) that uncovers networks that can produce a particular set of cell types, but it does not necessarily find the actual biological network.

We modeled the simplified myeloid lineage network that is shown in Figure 1 to demonstrate the utility of the visualization technique. The modeling was based on the work of Krumsiek et al. [4] who considered a network of eleven transcription factors known to be important in myeloid cell differentiation. We extended this work by applying a novel search technique (paper in preparation) to discover a new Boolean regulatory network that is supported by the literature and whose dynamics produce all the attractors in the lineage tree, three attractors representing pluripotent cells, along with an additional 4 attractors representing the terminally differentiated cell types. The transcription factor expression pattern of each of these attractors corresponds to a myeloid cell type shown in Figure 1. Our GRN discovery method searches the space of Boolean GRNs converging to a specific GRN that minimizes the difference between the attractor's Boolean expression values and the experimental expression values of the corresponding cell types. The new inferred Boolean GRN is illustrated in Figure 3. The essential point for demonstrating the value of the* CellDiff3D* approach is that this network produces transitions between cell types that cannot be visualized using Waddington's epigenetic landscape or conventional lineage trees but can easily be seen and analyzed using* CellDiff3D*.

**Figure 4:**
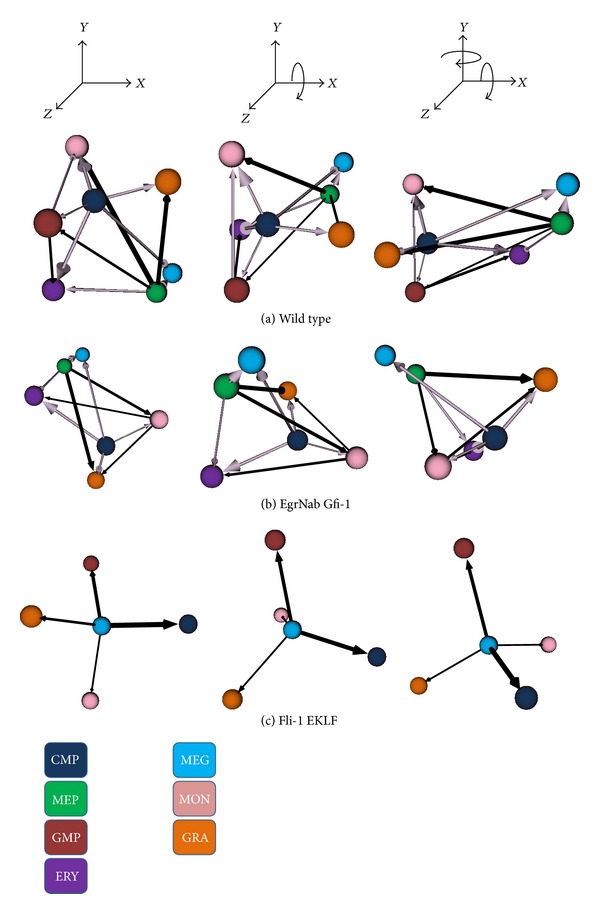
*CellDiff3D* visualization of the simulated myeloid differentiation network. Each image is a still taken from renderings of VRML code produced by the modeling method. The transcription factors and their regulatory interactions that comprise the GRN are shown in Figure 3. Each sphere is one of the myeloid cell types shown in Figure 1. Each row shows three orthographic views of cell type transitions derived from runs using the wild-type transcription factor network (top row of panel) or with transcription factor mutations in which the first transcription factor listed does not interact with the second transcription factor (lower rows of panel). The distance between each pair of cell types is the separation and the arrow direction and thickness are flux. For clarity, low flux edges are not shown. Lavender arrows show normal differentiation or dedifferentiation along the standard lineage tree from a specialized cell to its immediate progenitor; black arrows show transdifferentiation, off-differentiation, or off-dedifferentiation.

Figure 4 shows some outputs of the visualization method applied to simulated myeloid differentiation GRN. Running the myeloid GRN resulted in four attractors with gene expression levels that closely match the four terminally differentiated cell types (erythrocytes (ERY), megakaryocytes (MEG), monocytes (MON), and granulocytes (GRA)). In addition, there are three attractors that correspond to the MEP and GMP progenitors and the CMP stem cell (expression data is given in [4]).

**Figure 5:**
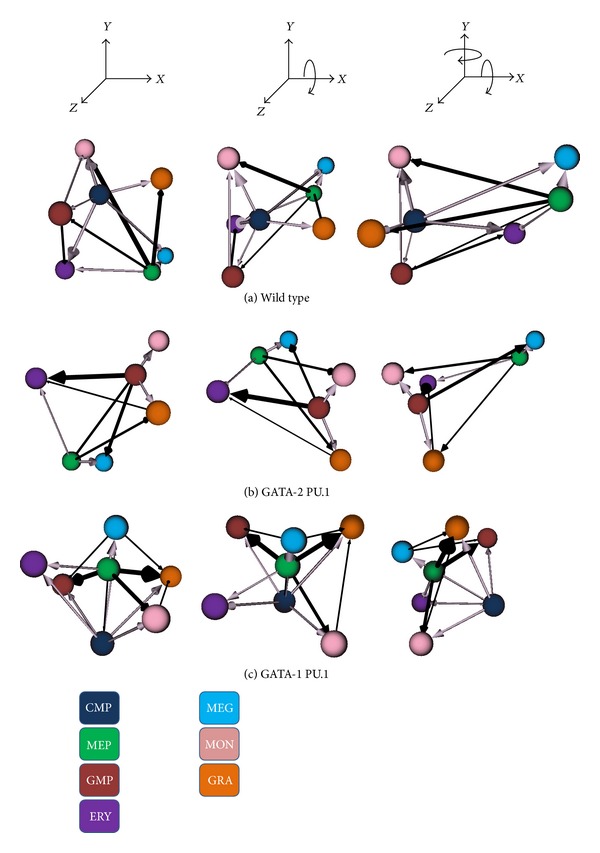
*CellDiff3D* illustration of the effects of two additional mutations that disrupt the myeloid differentiation network. There are interactions between GATA-2 to PU.1 (middle row) and GATA-1 to PU.1 (bottom row). See Figure 4 for extended caption.

Each row of Figures 4 and 5 shows three different orthographic projections of the 3D graph of the attractor network. The inferred Boolean network generated the seven stable attractors produced during normal myeloid differentiation (labeled wild type in Figures 4 and 5). Rows below the wild-type network show how network modifications (equivalent to mutations) alter the attractor landscape and how the technique described here can readily visualize these changes. These mutated GRNs were created by knocking out the forward interaction link between a transcription factor and one of its targets by always assigning this link a value of false then running the network to compute the MFPT. For example, in the second row of Figure 4, we fix the value of the link from transcription factor EgrNab to transcription factor Gfi-1 in the network shown in Figure 3.

A key point in interpreting the visualized lineage networks is understanding flux and separation. For example, in the wild-type network of Figure 4, note the wide spacing between the granulocyte (GRA; orange) and megakaryocyte-erythrocyte precursor (MEP; green) cells and the narrowness of the arrow that connects these cells. The large distance indicates that there is a low probability for this cell type transition, the direction of the arrow shows the overall direction of this infrequent transition, and the narrow width of the arrow indicates that there is relatively little difference between the forward and reverse rates of the transitions between these cells. Therefore, this is an infrequent and low flux transition. Similarly, the wide separation and lack of an arrow (signalling a very low flux) indicate that granulocyte (GRA; orange) and monocyte (MON; pink) terminal differentiation is stable and transdifferentiation is rare.

Contrast this with the arrow connecting the monocytes (MON; pink) and common myeloid precursor (CMP; dark blue) cells shown in the same row of the figure. The separation between these cell types is small, indicating a low MFPT and a high probability of this transition and the thick arrow connecting the CMP to the MON cells indicates the overall direction of the cell state transition (CMP to MON) and that the rate of the CMP to MON forward transition far exceeds the rate of the reverse transition. Therefore, this is a frequent and high flux transition. The ability to rotate this graph freely using the VRML viewer tool adds to the utility of the visualization as the viewer can explore the relationships between all pairs of cell types within this, or any other, lineage network.

Comparisons of the wild-type network with mutated networks in which one of the interactions between transcription factors is blocked reveal strong differences in lineage network organization. For instance, in the bottom panel of Figure 4, our visualization method immediately demonstrates major alterations in the lineage tree due to blocking Fli-1′s regulation of EKLF. In this case, two cell types, megakaryocyte-erythrocyte progenitor (MEP) and erythrocytes (ERY), are no longer present.

Finally, the technique developed here is able to reveal many different kinds of transitions between cell states (Table 2). Although a GRN that produces attractors that correspond to myeloid cell types was used in this initial study, any GRN and its resulting attractors/cell types can be explored using this approach. Significantly, nonstandard transitions, such as dedifferentiation, off-differentiation, and transdifferentiation, are increasingly recognized in normal and disease states, many of which cannot be shown using conventional lineage trees. Our method allows their representation in 3-dimensional space and provides important information on their likelihood under either gene expression noise as shown here, or other driving forces in GRN dynamics.

## 3. Methods

### 3.1. Cell Differentiation and Attractor Dynamics

First proposed by Kauffman [17], Boolean networks are one of the main contributors to our current knowledge of gene regulatory networks. They have proved effective in representing many biological systems including* Drosophila* development [21, 22], angiogenesis [23], eukaryotic cell dynamics [24], and yeast transcription networks [25]. Boolean networks consist of nodes and directed edges. In GRN modeling, nodes represent the genes and edges represent the regulatory influences between the genes. These regulatory influences are fully defined by the updating rules for each gene as a logic function of the inputs. A gene can be either expressed (the output is true) or not expressed (the output is false).

A Boolean network with *n* genes has 2^*n*^ possible states, denoted as $\hat{S}$. Each network state ${\hat{s}}_{t}$ is the collection of all gene values at time *t*, ${\hat{s}}_{t}=\{g_{1},g_{2},\ldots ,g_{n}\}$. Given the current state ${\hat{s}}_{t}$, the next network state ${\hat{s}}_{t+1}$ is obtained by applying each gene's function to the the current gene values. The gene's logic functions are deterministic. Thus, the the mapping function $D({\hat{s}}_{t})$ that finds the next network state is also deterministic: ${\hat{s}}_{t+1}\leftarrow D({\hat{s}}_{t})$. By repeatedly applying deterministic updating, the network dynamics will eventually reach a previously visited state. This cycle is called an attractor ($\hat{a}$). Attractors can be single states, called point attractors or cyclic attractors in which the cycle consists of more than one state. Note that to find all attractors of a given network all possible starting states need to be considered (the code can be obtained from http://code.google.com/p/pbn-matlab-toolbox).

In this work, cell types are considered attractors in the state space of possible gene expression profiles [26] and cell differentiation is modeled as the process of transitioning from one attractor to another [6].

### 3.2. Simulating and Measuring Noise Dynamics

Noise at the molecular level plays a key role in many biological processes including protein folding, transcription factor binding to DNA, and the rate of initiating transcription and translation [27, 28]. At the systems level, noise influences the likelihood of cell type transitions [26]. Noise can be modeled in Boolean regulatory networks by random bit flips during network operation, with these bit flips representing noise-driven changes in gene expression. Let ${\hat{s}}_{j}\leftarrow \eta ({\hat{s}}_{i},r)$ be the spontaneous noise function that maps a state of the network ${\hat{s}}_{i}$ to a new state ${\hat{s}}_{j}$ with the addition of noise, implemented as *r* bit flips, with each single bit flip occurring with probability *p*. Noise modifies the probability of state transitions as the states are updated and the switching among network attractors. Since attractors represent cell types, measures of noise tolerance can estimate the magnitude of the barrier between attractors, the so-called epigenetic barrier. In the following section, three measures of the epigenetic barrier are introduced and compared.

#### 3.2.1. Hamming Distance

Hamming distance is the direct measure of the difference between corresponding elements of two bit vectors. In GRNs, Hamming distance measures the differences in expression levels between two network states. Differences between gene expression profiles are used to identify cell type or cell physiology [29]. However, as a measure of the epigenetic barrier between states, Hamming distance does not utilize $\eta (\hat{s},r)$ and also ignores the constraints that regulatory network dynamics impose upon state transitions $D(\hat{s})$. For these reasons, Hamming distance is a poor measure of the epigenetic barrier.

#### 3.2.2. Transitory Perturbation (Single-Bit-Flip)

An alternative measure of the likelihood of attractor transition under expression noise was introduced by Villani et al. [30]. Once the set of attractors is identified, this measure inserts noise as a single bit flip one-off event followed by deterministic updating. So given ${\hat{a}}_{i}$ as an attractor state, ${\hat{s}}_{i}\leftarrow \eta ({\hat{a}}_{i},1)$ is applied to a single bit, and then the network defined updating rules are applied determinatively until an attractor state ${\hat{a}}_{j}\leftarrow D^{*}({\hat{s}}_{i})$ is reached. For each attractor and each bit, the process is repeated. Let *c*_*i*,*j*_, 1 ≤ *i*, *j* ≤ *m* (where *m* is the number of attractors), be the count of cases when ${\hat{a}}_{j}\leftarrow D^{*}(\eta ({\hat{a}}_{i},1))$. Then, $P({\hat{a}}_{i},{\hat{a}}_{j})=c_{i,j}/m$. For each pair of attractors $\left\{{\hat{a}}_{i},{\hat{a}}_{j}\right\}$, $P({\hat{a}}_{i},{\hat{a}}_{j})$ is the portion of single one-step bit flips (transitory perturbations) in the nodes of all states of attractor ${\hat{a}}_{i}$ which will result in a transition from ${\hat{a}}_{i}$ to ${\hat{a}}_{j}$ under noise-free dynamics.

This single-bit-flip measure of likelihood of network transition under noise efficiently estimates the epigenetic barrier (since it is *O*(*nm*)), but it assumes that expression noise is an infrequent event during network dynamics.

### 3.3. Mean First Passage Time

Introduced by Shmulevich et al. [16], mean first passage time (MFPT) is the the average time it takes to reach state *y* from state *x* in the presence of noise. Mathematically, first passage time (FPT) is defined as $F_{k}({\hat{s}}_{x},{\hat{s}}_{y})$, the probability that starting in state ${\hat{s}}_{x}$; the first time the system visits a state ${\hat{s}}_{y}$ will be at time *k*; in Boolean networks, time is measured as the number of state updates. MFPT is then defined as

(4) $$\begin{array}{c} \text{MFPT}\left({\hat{s}}_{x},{\hat{s}}_{y}\right)=\underset{k}{\sum }kF_{k}\left({\hat{s}}_{x},{\hat{s}}_{y}\right), \end{array}$$

where the *F*_*k*_ itself is formulated as

(5) $$\begin{array}{c} F_{k}\left({\hat{s}}_{x},{\hat{s}}_{y}\right)=\underset{{\hat{s}}_{z}\in {\left\{0,1\right\}}^{n},z\neq y}{\sum }p_{xz}F_{k-1}\left({\hat{s}}_{z},{\hat{s}}_{y}\right). \end{array}$$

In this recursive formula, $F_{1}({\hat{s}}_{x},{\hat{s}}_{y})$ is the probability of direct transition from state ${\hat{s}}_{x}$ to ${\hat{s}}_{y}$. *p*_*xz*_ is the probability of transition from state ${\hat{s}}_{x}$ to state ${\hat{s}}_{z}$. Probabilistically, there are two ways to reach state ${\hat{s}}_{z}$ from ${\hat{s}}_{x}$; either ${\hat{s}}_{z}$ is a deterministic target for ${\hat{s}}_{s}$ and no bit flips occur due to the noise or an aggregate of bit flips drives the transition from ${\hat{s}}_{x}$ to ${\hat{s}}_{z}$.

**Figure 6:**
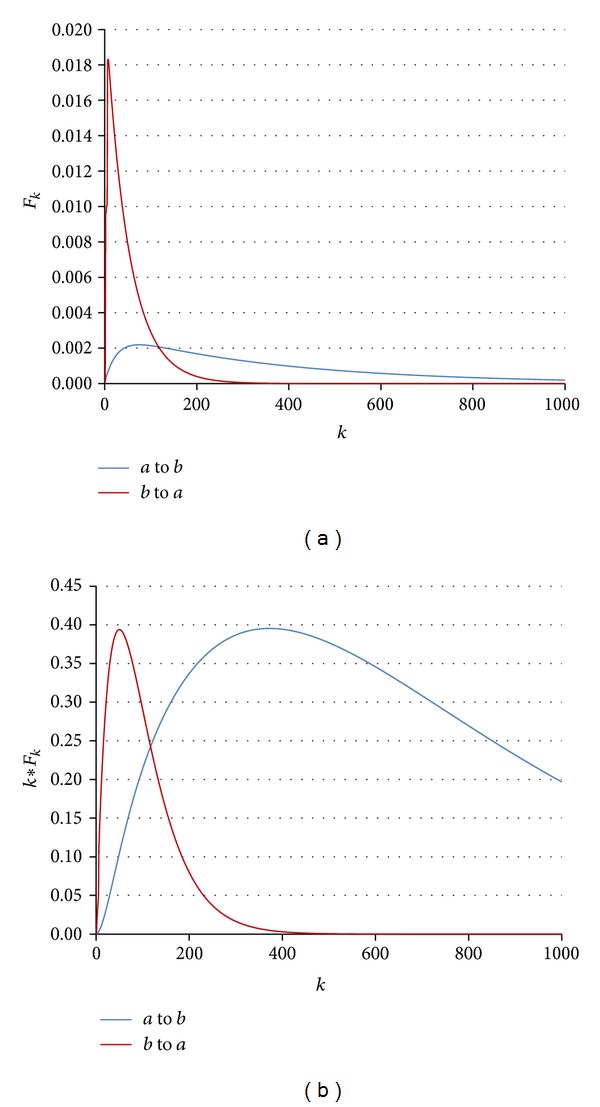
(a) *F*_*k*_ (probability of first visit at time step *k*) plotted for two arbitrary attractors, called *a* and *b* in a random Boolean network for 1000 steps (*k*). The red curve is for the transition from *b* to *a* that has a low MFPT compared to the reverse transition; *a* to *b* is shown with the blue curve; (b) *kF*_*k*_ plotted for the *F*_*k*_ curves in (a). Note that MFPT is the centroid of the area under the *kF*_*k*_ curve.

When the MFPT between two states is low, it implies that, starting from the first state, the second state is easily reached by molecular noise.  Figure 6 shows *F*_*k*_ and *kF*_*k*_ for the transition between two arbitrary attractors. As this figure shows, the *b* to *a* transition has a lower MFPT compared with *a* to *b*. Note that when an attractor has more than one state; that is, it is a cyclic attractor, the MFPT is calculated for each state separately and then is averaged over all states of that attractor.

At each network state update $D(\hat{s})$ there is a probability that the state will change as a function of the Hamming distance (*h*) between the current state and the subsequent state ${\hat{s}}_{t+1}\leftarrow D(\eta ({\hat{s}}_{t},r))$. MFPT models uniform expression noise by considering probabilistic bit flips at every possible state of the network and deriving the distribution of passage times from analysis of the corresponding Markov process. Statistically, the probability distribution of bit flips can be seen as a binomial distribution, and thus the probability of *r* bit flips, $\eta ({\hat{s}}_{a},r)$, is $(\begin{array}{c} n\\ r \end{array})p^{r}{\left(1-p\right)}^{n-r}$, where *p* is the probability of a single bit flip and *n* is the total number of bits.

Mean first passage time quantifies the epigenetic barriers between all attractor states during network execution. Therefore, this work only considers MFPT because of its realism in modeling expression noise. However, the time required for MFPT computation is an exponential function of the number of genes, so if the number of genes in the network is large, calculating MFPT may become intractable. In this case, transitory perturbation can be used as a possible alternative.

## 4. Summary

In this work, we developed a technique and a supporting method for visualization,* CellDiff3D*, that estimates the likelihood and directionality of noise-driven transitions between different cell types and allows the three-dimensional visualization of these relationships. A Boolean network model of myeloid cell differentiation [4] was used as a demonstration system for this research.

The metric of mean first passage time (MFPT) assesses the likelihood that noise in the GRN for myeloid differentiation will trigger a transition between cell types. Low MFPT values indicate a high probability of a cell type transition. The difference in MFPTs for forward (cell type A to cell type B) and reverse (cell type B to cell type A) transitions provides a measure termed flux. Flux is analogous to the difference in forward and reverse rates of a chemical reaction and it gives the anticipated direction and the strength of the directionality in transitions between cell types.

Our technique calculated the MFPT separation and flux between all pairs of cell types in a simplified myeloid lineage tree that included one multipotent stem cell, two intermediate cells, and four terminal cell types to produce a graph to display all 42 pairwise relationships *m*(*m* − 1) where *m* = 7 between the myeloid cell types. A VRML-based graphics tool was employed as part of* CellDiff3D* to visualize all attractor type transitions by placing all pairs of different cell types in 3-dimensional space. It shows the likelihood of a transition between cell types as the separation between each pair and the directionality of the transition as arrows with a width proportional to the flux. The VRML output, viewable in any web browser (with the proper plugin), allows the free rotation and zooming of the differentiation network to reveal its features. It can be used for any cell differentiation network, can include many more than the 7 cell types considered here, and is capable of showing all possible transitions (e.g., dedifferentiation and transdifferentiation) between different types of cells. Our technique readily revealed changes in the dynamics of mutationally altered myeloid differentiation networks, the loss of cell types, and unusual cell type transitions that included dedifferentiation, transdifferentiation, and off-differentiation.

This work has introduced a 3D graph approach to visualize the influence of noise on cell type switching of wild-type and mutated regulatory networks. However, the system is not limited to noise analysis and can incorporate other influences that drive cell type switching. The* CellDiff3D* tool can be downloaded from http://www.celldiff3d.org/.

## Figures and Tables

**Table 1:** Summary of different kinds of cell type transitions with possible examples from myeloid differentiation tree shown in Figure 1.

Transition	Example	Definition
Spontaneous-differentiation	CMP to MEP	Cell switches to a more specialized state
Spontaneous-dedifferentiation	MON to GMP	Cell reverts to an earlier multipotent state
Off-differentiation	GMP to ERY	Cell switches to a more specialized state but on a wrong branch of the lineage tree
Off-dedifferentiation	MEG to GMP	Differentiated cell reverts to an earlier multipotent state but on a wrong branch of the lineage tree
Transdifferentiation	GRA to ERY	Differentiated cell switches to another differentiated state

**Table 2:** Cell type transitions discovered and visualized in the myeloid differentiation network shown Figure 3 and in mutationally altered forms of this network.

Figure	Network	Cell type switch	Kind
Figure 4(a)	Wild type	CMP ⇒ MON	Spontaneous-differentiation
		MEP ⇒ GMP	Off-differentiation
		MEG ⇔ CMP	High separation
		MEP ⇒ GMP	Off-differentiation
		MEP ⇔ MEG	Low separation

Figure 4(b)	EgrNab/Gfi-1	CMP ⇒ MEG	Spontaneous-differentiation
		ERY ⇒ GRA	Transdifferentiation
		GRA ⇔ CMP	Low separation

Figure 4(c)	Fli-1/EKLF	MEG ⇒ CMP	Spontaneous-dedifferentiation
		MEG ⇒ MON	Transdifferentiation
		MEG ⇒ GMP	Off-differentiation
		MEG ⇔ MON	Low separation
		GMP ⇔ MON	High separation

Figure 5(b)	GATA-2/PU.1	GRA ⇒ CMP	Spontaneous-dedifferentiation
		MEP ⇒ MON	Off-differentiation
		GMP ⇒ ERY	Off-differentiation
		GMP ⇒ MEG	Off-differentiation

Figure 5(c)	GATA-1/PU.1	MON ⇒ GMP	Transdifferentiation

## References

[1] Huang S (2009). Reprogramming cell fates: reconciling rarity with robustness. *BioEssays*.

[2] Masaki T, Qu J, Cholewa-Waclaw J, Burr K, Raaum R, Rambukkana A (2013). Reprogramming adult Schwann cells to stem cell-like cells by Leprosy Bacilli promotes dissemination of infection. *Cell*.

[3] Their JP (2002). Epithelial-mesenchymal transitions in tumor progression. *Nature Reviews Cancer*.

[4] Krumsiek J, Marr C, Schroeder T, Theis FJ (2011). Hierarchical differentiation of myeloid progenitors is encoded in the transcription factor network. *PLoS ONE*.

[5] Huang S, Eichler G, Bar-Yam Y, Ingber DE (2005). Cell fates as high-dimensional attractor states of a complex gene regulatory network. *Physical Review Letters*.

[6] Chang HH, Hemberg M, Barahona M, Ingber DE, Huang S (2008). Transcriptome-wide noise controls lineage choice in mammalian progenitor cells. *Nature*.

[7] Orrell D, Bolouri H (2004). Control of internal and external noise in genetic regulatory networks. *Journal of Theoretical Biology*.

[8] Huang S, Ernberg I, Kauffman S (2009). Cancer attractors: a systems view of tumors from a gene network dynamics and developmental perspective. *Seminars in Cell and Developmental Biology*.

[9] MacNeil LT, Walhout AJM (2011). Gene regulatory networks and the role of robustness and stochasticity in the control of gene expression. *Genome Research*.

[10] Hu Q, Friedrich AM, Johnson LV, Clegg DO (2010). Memory in induced pluripotent stem cells: reprogrammed human retinal-pigmented epithelial cells show tendency for spontaneous redifferentiation. *Stem Cells*.

[11] Waddington CH (1957). *The Strategy of Genes*.

[12] Furusawa C, Kaneko K (2012). A dynamical-systems view of stem cell biology. *Science*.

[13] Suzuki N, Furusawa C, Kaneko K (2011). Oscillatory protein expression dynamics endows stem cells with robust differentiation potential. *PLoS ONE*.

[14] Zhou JX, Aliyu MDS, Aurell E, Huang S (2012). Quasi-potential landscape in complex multi-stable systems. *Journal of the Royal Society Interface*.

[15] Bhattacharya S, Zhang Q, Andersen M (2011). A deterministic map of Waddington’s epigenetic landscape for cell fate specification. *BMC Systems Biology*.

[16] Shmulevich I, Dougherty ER, Zhang W (2002). Gene perturbation and intervention in probabilistic Boolean networks. *Bioinformatics*.

[17] Kauffman SA (1969). Metabolic stability and epigenesis in randomly constructed genetic nets. *Journal of Theoretical Biology*.

[18] Xue Y, Ouyang K, Huang J (2013). Direct conversion of fibroblasts to neurons by reprogramming PTB-regulated MicroRNA circuits. *Cell*.

[19] Vierbuchen T, Ostermeier A, Pang ZP, Kokubu Y, Südhof TC, Wernig M (2010). Direct conversion of fibroblasts to functional neurons by defined factors. *Nature*.

[20] Ellson J, Gansner ER, Koutsofios E, North SC, Woodhull G (2003). Graphviz and dynagraph—static and dynamic graph drawing tools. *Graph Drawing Software*.

[21] Albert R, Othmer HG (2003). The topology of the regulatory interactions predicts the expression pattern of the segment polarity genes in Drosophila melanogaster. *Journal of Theoretical Biology*.

[22] Bodnar JW (1997). Programming the *Drosophila* Embryo. *Journal of Theoretical Biology*.

[23] Bauer AL, Jackson TL, Jiang Y, Rohlf T (2010). Receptor cross-talk in angiogenesis: mapping environmental cues to cell phenotype using a stochastic, Boolean signaling network model. *Journal of Theoretical Biology*.

[24] Shmulevich I, Kauffman SA, Aldana M (2005). Eukaryotic cells are dynamically ordered or critical but not chaotic. *Proceedings of the National Academy of Sciences of the United States of America*.

[25] Kauffman S, Peterson C, Samuelssont B, Troein C (2003). Random Boolean network models and the yeast transcriptional network. *Proceedings of the National Academy of Sciences of the United States of America*.

[26] Huang AC, Hu L, Kauffman SA, Zhang W, Shmulevich I (2009). Using cell fate attractors to uncover transcriptional regulation of HL60 neutrophil differentiation. *BMC Systems Biology*.

[27] Bar-Even A, Paulsson J, Maheshri N (2006). Noise in protein expression scales with natural protein abundance. *Nature Genetics*.

[28] Tkačik G, Gregor T, Bialek W (2008). The role of input noise in transcriptional regulation. *PLoS ONE*.

[29] Bruno L, Hoffmann R, McBlane F (2004). Molecular signatures of self-renewal, differentiation, and lineage choice in multipotential hemopoietic progenitor cells in vitro. *Molecular and Cellular Biology*.

[30] Villani M, Barbieri A, Serra R (2011). A dynamical model of genetic networks for cell differentiation. *PLoS ONE*.

